# Limited Microcystin, Anatoxin and Cylindrospermopsin Production by Cyanobacteria from Microbial Mats in Cold Deserts

**DOI:** 10.3390/toxins12040244

**Published:** 2020-04-11

**Authors:** Nataliia Khomutovska, Małgorzata Sandzewicz, Łukasz Łach, Małgorzata Suska-Malawska, Monika Chmielewska, Hanna Mazur-Marzec, Marta Cegłowska, Toirbek Niyatbekov, Susanna A. Wood, Jonathan Puddick, Jan Kwiatowski, Iwona Jasser

**Affiliations:** 1 Institute of Environmental Biology, Faculty of Biology, Biological and Chemical Research Centre, University of Warsaw, Żwirki i Wigury 101, 02-089 Warsaw, Poland; n.khomutovska@uw.edu.pl (N.K.); sandzewicz@biol.uw.edu.pl (M.S.); lukaszlach@biol.uw.edu.pl (Ł.Ł.); malma@biol.uw.edu.pl (M.S.-M.); m.karbowa@uw.edu.pl (M.C.); jmkwiato@biol.uw.edu.pl (J.K.); 2 Division of Marine Biotechnology, Institute of Oceanography, University of Gdańsk, Marszałka Piłsudskiego 46 ave., 81-378 Gdynia, Poland; hanna.mazur-marzec@ug.edu.pl; 3 Department of Chemistry and Biochemistry, Institute of Oceanology, Polish Academy of Sciences, Powstańców Warszawy 55, 81-712 Sopot, Poland; mceglowska@iopan.gda.pl; 4 Institute of Botany, Plant Physiology and Genetics, Academy Science Republic of Tajikistan, 27 Karamov Str., Dushanbe 734017, Tajikistan; tohir-73@mail.ru; 5 Cawthron Institute, Private Bag 2, Nelson 7042, New Zealand; Susie.Wood@cawthron.org.nz (S.A.W.); Jonathan.Puddick@cawthron.org.nz (J.P.)

**Keywords:** cyanotoxins, microbial mats, V3-V4 hypervariable region, 16S rRNA gene, mcyA, mcyE, mcyD, ndaF, anaC, sxtA, potential toxin producers

## Abstract

Toxic metabolites are produced by many cyanobacterial species. There are limited data on toxigenic benthic, mat-forming cyanobacteria, and information on toxic cyanobacteria from Central Asia is even more scarce. In the present study, we examined cyanobacterial diversity and community structure, the presence of genes involved in toxin production and the occurrence of cyanotoxins in cyanobacterial mats from small water bodies in a cold high-mountain desert of Eastern Pamir. Diversity was explored using amplicon-based sequencing targeting the V3-V4 region of the 16S rRNA gene, toxin potential using PCR-based methods (*mcy, nda, ana, sxt*), and toxins by enzyme-linked immunosorbent assays (ELISAs) and liquid chromatography-tandem mass spectrometry (LC-MS/MS). Molecular identification of cyanobacteria showed a high similarity of abundant taxa to *Nostoc* PCC-73102, *Nostoc* PCC-7524, *Nodularia* PCC*-*935 and *Leptolyngbya* CYN68. The PCRs revealed the presence of *mcyE* and/or *ndaF* genes in 11 samples and *mcyD* in six. The partial sequences of the *mcyE* gene showed high sequence similarity to *Nostoc*, *Planktothrix* and uncultured cyanobacteria. LC-MS/MS analysis identified six microcystin congeners in two samples and unknown peptides in one. These results suggest that, in this extreme environment, cyanobacteria do not commonly produce microcystins, anatoxins and cylindrospermopsins, despite the high diversity and widespread occurrence of potentially toxic taxa.

## 1. Introduction

Microbial mats are multi-structural communities comprised of eukaryotic algae, fungi, bacteria, including cyanobacteria and archaea, which form complex biofilms [1,2]. The mats are found from the equator through the temperate zones, and in the North and South poles. They vary in taxonomic composition and structure, which determines their shape and morphology [2,3,4]. Cyanobacteria play a significant role in these microbial mat communities due to their ability to conduct photosynthesis, fix atmospheric nitrogen and produce extracellular polysaccharides. Thus, they provide organic carbon and biologically active nitrogen, and help to maintain the shape and structure of the microbial mats. Production of scytonemin and mycosporine-like amino acids by cyanobacteria help to protect them from UV radiation [5,6]. They also produce many other bioactive compounds [7], which are believed to increase their chances of surviving in inhospitable environments, such as extremely cold or hot deserts and hypersaline habitats [3,4,8].

Some cyanobacteria also produce cyanotoxins, which pose a health hazard for humans, wildlife and domestic animals [9,10]. The factors promoting the production and the ecological function of cyanotoxins in water and terrestrial habitats are unclear [11,12,13,14]. Cyanotoxins can be divided into five main classes according to their main targets in toxicity: hepatotoxins (e.g., microcystins; MCs), neurotoxins (e.g., anatoxins and saxitoxins), dermatoxins, cytotoxins (e.g., cylindrospermopsins) and lipopolysaccharides (irritant toxins) [7,15,16]. Among them, microcystins are the most commonly identified in lakes and reservoirs [7,17].

Almost all cyanotoxins known from planktonic environments have also been detected in benthic mats from various temperate and subtropical conditions; however, little is known about cyanotoxins in cold ecosystems [18,19,20]. Kleinteich et al. [3,4,21] and Wood et al. [22] studied waterbodies close to both magnetic poles and reported the presence of microcystins in Arctic and Antarctic microbial mats [3,4,21] and saxitoxin [3] in benthos from the Arctic, cylindrospermopsin in the Antarctic [4], as well as [Asp^3^, ADMAdda^5^, Dhb^7^] MC-LR congeners in Svalbard [21]. 

However, most of the data concerning production of cyanotoxins (as well as studies related to genes encoding toxin production) have come from planktonic cyanobacteria [23]. Thus, many of the highly selective analytical methods (e.g., liquid chromatography-tandem mass spectrometry multiple reaction monitoring methods; LC-MS/MS MRM) for microcystin detection may be biased towards the congeners commonly observed in planktonic cyanobacteria. 

The taxonomic composition of microbial mats is different from that of planktonic samples and there is uncertainty as to whether universal primers, which were designed for planktonic species, can be applied for the identification of these genes in microbial mats. Bukowska et al. [24] noted that using universal primers for microcystin-encoding genes designed for planktonic communities might be insufficient to identify these genes in many species and may lead to an underestimation of the presence of toxin-producing cyanobacteria in some environments.

The distribution of genes encoding toxin production and the presence of cyanotoxins is poorly known in most regions of Central Asia. Most studies concerning toxicology and ecotoxicology of cyanotoxins/cyanobacteria are from Europe, North and Central America (47%), while the studies from Asia provide only about 17% of the data [25]. There are only incidental data from Central Asia concerning toxigenic cyanobacteria and presence of cyanotoxins and no studies from Eastern Pamir (Tajikistan). Only a few studies are published on algae and cyanobacteria in plankton of Pamir’s lakes [26,27] and a single report of benthic cyanobacteria in Pamir’s wetlands [28].

**Figure 1:**
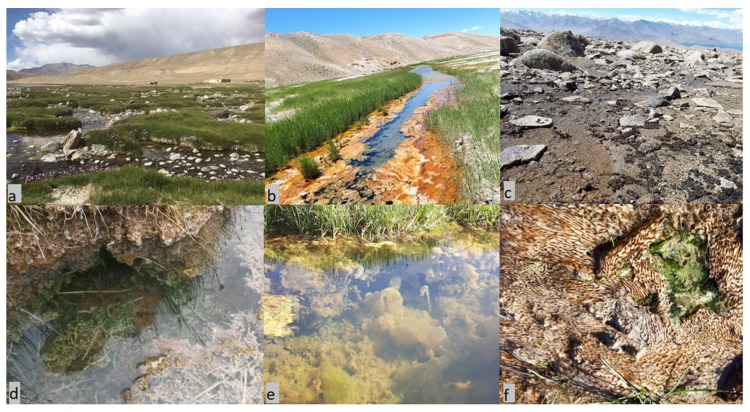
Distribution and diversity of microbial mats in Eastern Pamir. (**a**) Small waterbodies with microbial mats near Bulunkul Lake; (**b**) cyanobacterial mat in hot springs in the vicinity of Alichur village; (**c**) “*Nostoc*-type” microbial mats on the mountain peak 5006 m above sea level near Karakul Lake; (**d**) “Multilayer soft” type mats near Rangkul Lake; (**e**) “Jelly-like” microbial mat near Sassykkul Lake; and (**f**) “*Phormidium*-type” mat near Karakul Lake. Photo: a,d—I.J.; b,c,e,f—N.K.

This research was undertaken in Eastern Pamir in the Gorno-Badakhshan (Kuhistani Badakhshan) Autonomous Oblast (GBAO) of Tajikistan. Eastern Pamir is a high-mountain cold desert, which due to its isolation and harsh climatic condition [29,30] provides a unique environment for microbial growth. Microbial biofilms are widely distributed in Eastern Pamir and develop in a diverse range of water reservoirs such as small water bodies, pools, thermokarsts, ponds as well as hot springs. The mats also differ in thickness; they cover the bottoms or walls of the reservoir or float on the surface of the water (Figure 1). The lack of available water sources in rural areas in GBAO forces local people to use water from accessible water reservoirs, lakes, thermokarsts, streams and rivers. The study of cyanotoxins, as well as monitoring of cyanotoxins distribution in microbial mats occurring in GBAO is presently of great importance, because of the potential threat to humans and domestic and wild animals.

Our preliminary study concerning the composition and structure of benthic communities collected in 2014 showed the prevalence of Nostocales [28] that were greatly represented by commonly known toxin-producing genera such as *Nodularia* [31] and *Calothrix* [32]. Therefore, based on previous results, as well as data regarding toxin production by benthic Arctic and Antarctic communities [3,4,21] we expected the presence of at least hepatotoxins and cytotoxins in microbial mats. The aims of the present research were to: (1) analyze the diversity of cyanobacteria and identify potential toxigenic species, (2) test for the presence of genes involved in cyanotoxin production in the microbial mats, and (3) test for the presence of microcystins, anatoxins and cylindrospermopsins using biochemical and chemical methods. 

## 2. Results

### 2.1. Study Design

**Figure 2:**
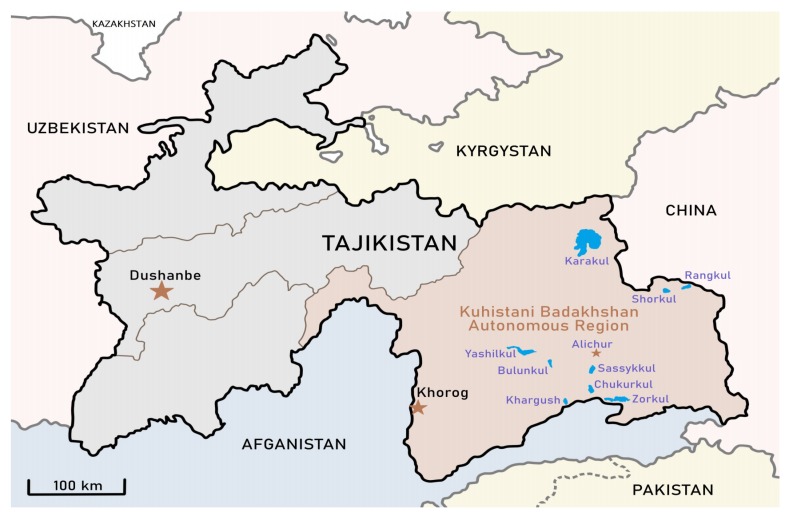
Investigated area located in Gorno-Badakhshan (Kuhistani Badakhshan) Autonomous Region of Tajikistan. The waterbodies situated near Pamirian lakes Yashilkul, Bulunkul, Sassykkul, Chukurkul, Khargush, Zorkul, Shorkul, Rangkul, Karakul and countryside named Alichur. Modified map from WikiMedia (https://commons.wikimedia.org).

The samples of microbial mats were collected from small waterbodies in wetlands around nine lakes in Eastern Pamir (Bulunkul, Sassykkul, Khargush, Chukurkul, Yashilkul, Rangkul, Shorkul, Zorkul, Karakul) and from a wetland close to Alichur village (Figure 2). Twenty-one samples were collected in July 2015 and thirty in July 2017. Sampled waterbodies from around a single lake were several hundred meters apart while the distance between lakes varied between several to >150 km. The waterbodies with visible microbial mats were sampled and similar types of microbial mats were collected from wetlands around all lakes when present (Supplementary Table S1). Overlaying water from selected sampling places was analyzed in situ by ELISA for microcystins, anatoxins and cylindrospermopsin. In the laboratory, microbial mat samples were analyzed for toxins with LC-MS/MS as well as screened for known toxin-encoding genes. As all 51 samples were analyzed by LC-MS/MS in the University of Warsaw; some of the samples from 2017 (12 and 16) were cross-checked in two other laboratories because of specificity of the studied material. The molecular and bioinformatic analyses with phylogenetic placement of the sequences obtained from NSG of V3-V4 hypervariable region of the 16S rRNA gene were performed on all 30 samples (E01-E30) from 2017.

### 2.2. Macro- and Microenvironmental Parameters of Studied Water Samples

Physical and chemical parameters differed between the samples, providing diverse conditions for microbial growth (Supplementary Table S2). This also complicated the detection of cyanotoxins because of the presence of inhibitors, such as the high content of extracellular polysaccharides as well as high concentration of Ca^2+^, Na^+^ ions. For several samples (E24, E29, E30), physical and chemical data were not acquired because they were collected from sampling sites that were almost dry. The samples E15 and E30 were collected from sites with the highest and lowest temperatures (47.7 °C and 10 °C respectively), while the highest pH was detected in the sampling sites where E04, E07, E12 and E13 were collected. The electrical conductivity (EC) ranged between 267 μS cm^−1^ (E10) and 58,300 μS cm^−1^ (E13). The concentration of organic carbon varied almost one-hundred-fold between 2.7 mg L^−1^ in the reservoir corresponding to sample E15 and to 186.9 mg L^−1^ in reservoir E07. The concentration of total nitrogen ranged between 0.27 mg L^−1^ (E10) and 19.04 mg L^−1^ (E04).

### 2.3. Distribution of Microbial Mats and Diversity of Mat-Forming Cyanobacterial Communities

In this study, we present the main types of microbial mat communities in 30 representative samples, focusing on potentially toxigenic taxa and population structures. A publication on the detailed taxonomic composition and structure of 51 communities sampled in Pamir is in preparation.

Based on their morphology, microbial mats were divided into seven types: (1) “jelly-like” is a soft gelatinous mat-like film; (2) “multilayer soft” indicates a structured mat composed of upper pale green or orange layer followed by brown, emerald-green and pink-reddish layers, sometimes ended with another emerald green layer; (3) “multilayer hard” is a mat consisting from at least two films and covered with mineral particles and calcified hard layer; (4) “*Phormidium*-type” is a smooth, homogenous bright green or greenish mat; (5) “*Phormidium* beneath soil” green biofilm covered with soil; (6) “*Nostoc*-type” wrinkled dark green biofilm; (7) “amorphous” which is a non-stratified, heterogonous, dark green mat (Figure 1, Supplementary Table S1).

**Figure 3:**
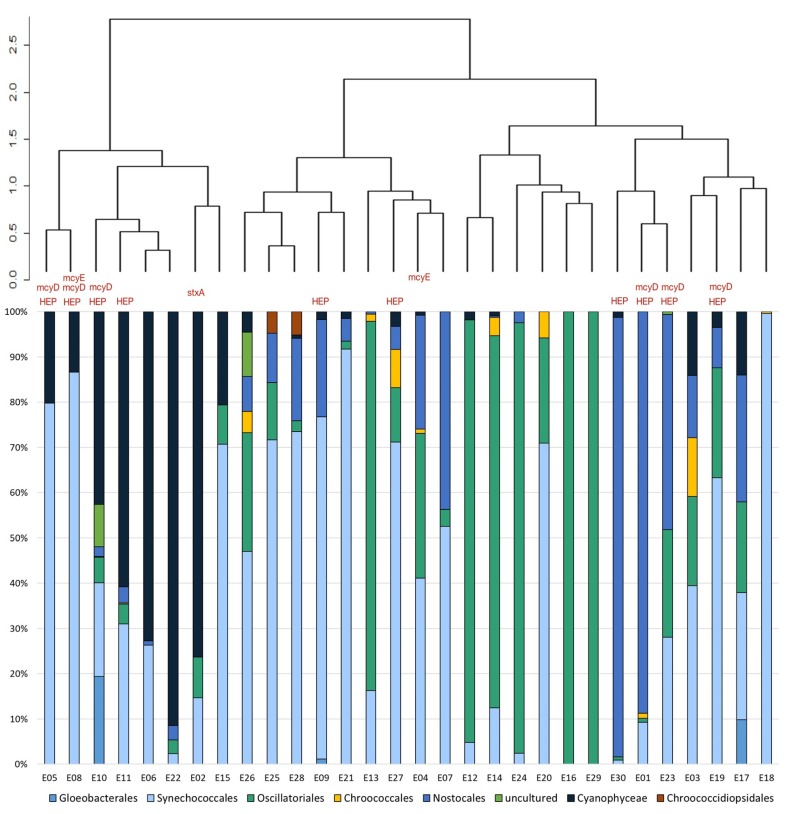
The structure of cyanobacterial communities, based on V3-V4 hypervariable region of 16S rRNA gene at genus level and distribution of genes encoding cyanotoxin production.

To study the composition and structure of the microbial mat communities, we performed 16S rRNA taxonomy assignment using SILVA 132 99% classifier in QIIME2, then filtered cyanobacterial sequences from the dataset and analyzed these separately. The total number of reads for the 2017 samples was 2,323,434 with an average of 77,500 reads per sample. The number of representative sequences obtained for cyanobacteria was 69,066, which are represented by 97 groups of amplicon sequence variants (ASVs). According to SILVA database (“silva-132–99-nb-classifier”) the sequence variants belong to orders Synechococcales (32,481; 47.1%), Nostocales (12,855; 18.6%), Oscillatoriales (10,868; 15.8%), Gloeobacterales (857; 1.2%), Chroococcales (633; 0.92%) and Chroococcidiopsidales (94; 0.14%). The remaining 16.33% of the ASVs were classified only to a phylum level, as Cyanophyceae (10,709; 15.5%) and as “uncultured” cyanobacteria (569; 0.83%; Figure 3).

There was no relationship between the structures of ASVs from closely located sampling sites. Synechococcales was the most diverse order, represented by 37 ASVs, while Oscillatoriales was divided into 27 ASVs. For Nostocales and Chroococcales we identified 11 ASVs for each order. The Gloeobacterales was the least diverse order represented by two ASVs, while Chroococcidiopsidales was represented by only one cluster of sequence variants. This was verified using a PaPaRa algorithm and phylogenetic placement method that allowed identifying them to Cyanophyceae only. We also obtained three types of cyanobacterial sequences classified as “uncultured”.

**Figure 4:**
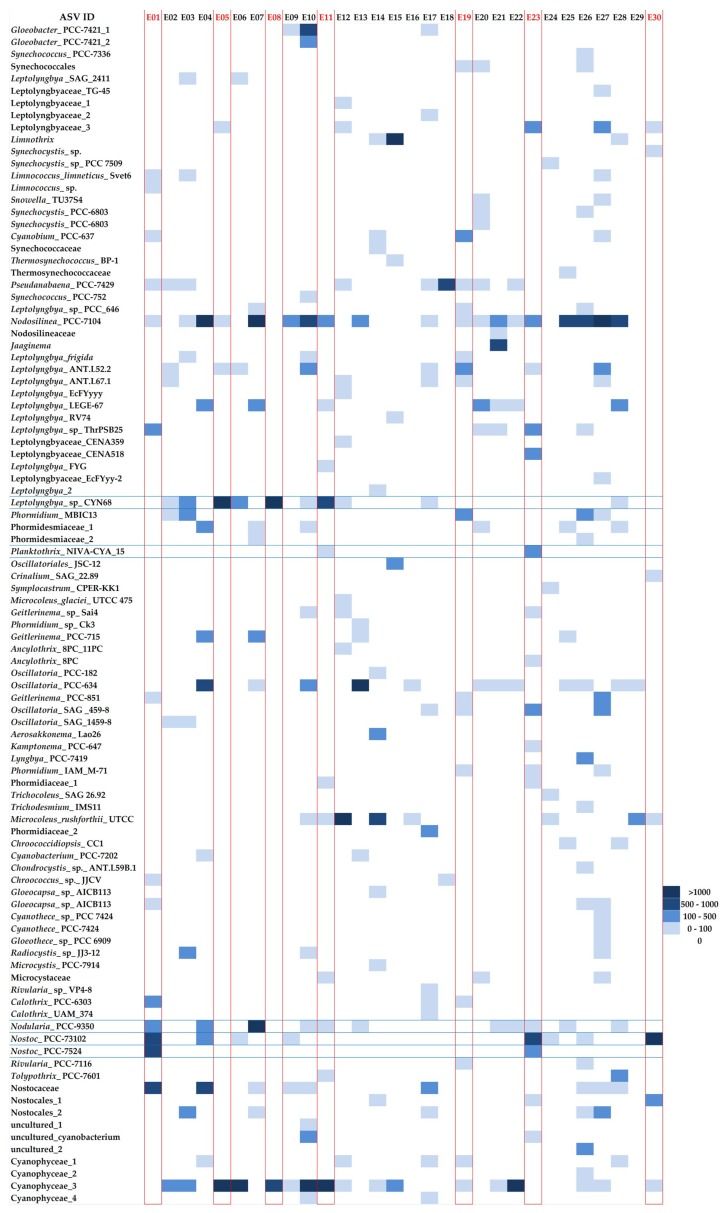
Heatmap of community structure based on V3-V4 hypervariable region of 16S rRNA gene. Samples, in which HEP primers amplified genes (*mcyE*/*ndaF*) were sequenced, are marked in red. The intensity of the blue color depicts the Amplicon Sequence Variants contribution in the samples, with dark blue showing the highest contribution.

The analysis of the ASV structure in studied microbial mats revealed a core community formed by sequences identical to *Nodosilinea* PCC-714, which were present in 19 mats (total number of ASVs 11,012), to *Oscillatoria* PCC-634, identified in 12 samples (2912 ASVs), and *Nodularia* PCC-935, from 11 samples with total of 4484 ASVs, and *Phormidium* CYN64 (10 samples, 14,527 ASVs). Some other sequences were also common but were found in less than 10 samples, among them were *Pseudanabaena* PCC-7429 (9 samples, 4333 ASVs), uncultured Antarctic cyanobacterium (9 samples, 1687 ASVs), *Nostoc* PCC-7312 (8 samples, 4484 ASVs), *Tychonema* CCAP 1459–11B (8 samples, 4058 ASVs) and *Leptolyngbya* LEGE-67 (7 samples, 757 ASVs; Figure 4).

### 2.4. Distribution of mcyA, mcyD, mcyE, mcyE/ndaF, sxtA, anaC genes in the Microbial Mat Samples and Detection of Potential Toxin Producers

The results of the PCR-based analysis performed to detect *mcyD* genes revealed their presence in seven samples (E05, E08, E10, E11, E19, E23, E30). These seven communities and three others (E01, E09, E27) also contained *mcyE* and/or *ndaF* genes amplified by HEP primers. Additionally, the *mcyE* gene amplified with mcyEF2 and mcyER4 primers were detected in one sample (E08), in which *mcyE* and/or *ndaF* genes amplified by HEP primers and *mcyD* genes were also present and in one sample in which no other microcystin-encoding genes were detected (E04). The *mcyA* genes were not detected in any sample. The *sxtA* gene was amplified only in one sample (sample E02). Despite many attempts with the *ana*C primers, we did not obtain amplicons of anatoxin-encoding genes (Figure 3, Supplementary Table S3).

The V3-V4 sequences identified for cyanobacteria were mapped on the Cydracil ML tree to verify the taxonomic position of abundant taxa as well as potential toxins producers. The placement of the sequences was further investigated and, where needed, the ASV names were corrected according to the phylogenetic position of the V3-V4 sequences. The phylogenetic tree showed that abundant ASVs classified as *Tychonema* CCAP 1459–11B were located on the *Microcoleus rushforthii* UTCC 296 branch (0.92 like weight ratio), while the cluster of *Phormidium* CYN64 sequences was situated on the *Leptolyngbya* sp. CYN68 branch with a like weight ratio of 1 (Supplementary, Figure S1). The sequences belonging to *Wilmottia* Ant-Ph58 were located on *Microcoleus glaciei* UTCC 475 branch, with a maximum weight ratio of 1. The sequences identified according to SILVA as *Nostoc* PCC 73102 were placed on *Calothrix* sp. CAWBG80 and *Nostoc* sp. UK18 with a weight ratio of 0.99 at both branches. Most of the sequences from *Aliterella* CENA595 ASVs cluster were located on *Synechocystis* sp. PCC 7509 branch (weight ratio of 1). The names of the sequences that mapped with like weight ratios lower than < 0.90 have not been changed. The adjustments were made prior to analysis at order level (Figure 3), thus order-clustering includes these changes.

To detect potential toxin producers, sequences amplified with HEP primers were analyzed using the BLAST and GenBank databases, as well as ML phylogenetic analysis. The HEP primer-amplified genes were detected in ten metagenomes (E01, E05, E08, E09, E10, E11, E19, E23, E27, E30) and good quality sequences were obtained from seven samples (E01, E05, E08, E11, E19, E23, E30). The BLAST analysis of sequences from these seven samples showed that sequences obtained from four samples (E05, E08, E8 culture, E11) were similar to *Planktothrix rubescens* NIVA-CYA 98 (95.7%, 94.2%, 94.6% and 95.9% respectively) with 92%–99% coverage. The sequence acquired from E01 (coverage—98%, identity—94.2%), E30 (coverage—91%, identity—95.4%) and E23 (coverage—64%, identity—93.2%) were similar to *Nostoc* sp. 152. The remaining sequence obtained from sample E19 was similar to uncultured cyanobacterium clone BaL 36–5/2015 aspartate aminotransferase (*mcyE*; coverage—90%, identity—91.2%).

The phylogenetic analysis confirmed the BLAST results showing that there were three distinct groups of *mcyE* genes. The group consisting of the sequences coming from samples E01, E23 and E30 formed a clade with sequences from *Nostoc* 152 and *Nodularia spumigena* UHCC 0039. The group consisting of sequences obtained from samples E05, E08 and E11, as well as from the E8 culture (single filament amplification obtained from the E08 mat) formed a sister clade with high support with *Planktothrix-Oscillatoria mcyE*-gene clade. The last sequence, obtained from sample E19, grouped with very high support (100) with sequences of aspartate aminotransferase (*mcyE*) gene, coming from uncultured cyanobacterium (clones BaL 33–3/2015, BaL 36–2/2015 and BaL 36–5/2015).

**Figure 5:**
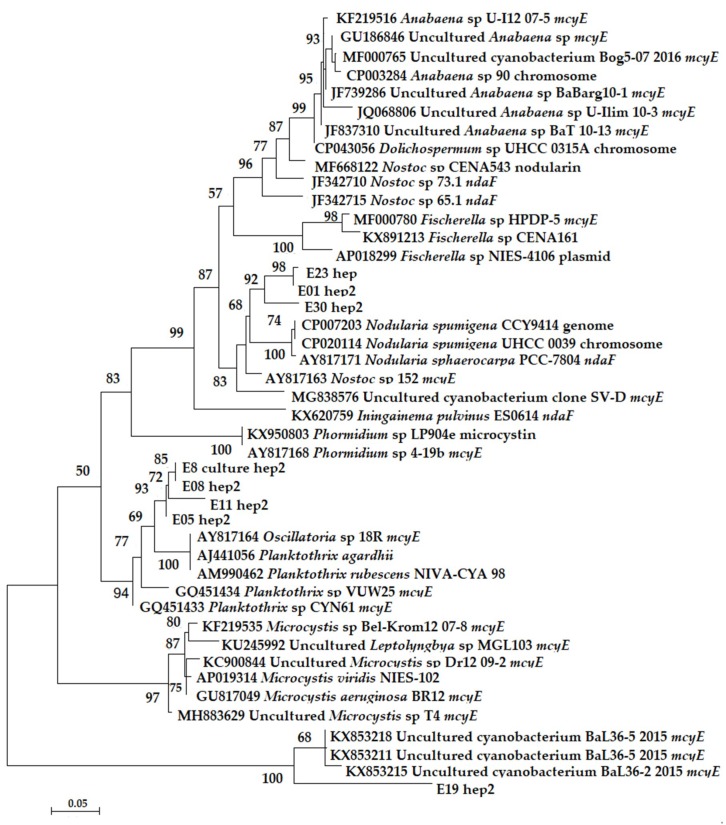
Maximum Likelihood phylogenetic tree of *mcyE*/*ndaF* gene sequences amplified with HEP primers for samples E01, E05, E08, E08 culture, E11, E19, E23 and E30. The acronym “hep” or “hep2” depicts amplification from which the sequences were used for the phylogenetic analysis.

The comparative analysis of V3-V4 sequences and phylogenetic analysis (Figure 4 and Figure 5) suggest that the *mc*y*E* genes detected in samples E01, E23, and E30 belonged to organisms closely related to *Nostoc* sp. and *Nodularia spumigena* UHCC 0039. Similarly, the *mcyE* genes detected in samples E05, E08 and E11, were from organisms closely related to *Planktothrix agardhii* NIVA-CYA, *Planktothrix rubescens* NIVA-CYA 98 and *Oscillatoria* sp. 18R. The sequence obtained from the sample E19 originated from the genomes of as-of-yet unidentified Cyanophyceae.

### 2.5. Detection of Cyanotoxins in Microbial Mats

Analysis for the presence of cyanotoxins in the overlaying water of benthic samples was performed using enzyme-linked immunosorbent assays (ELISAs) for microcystins, anatoxins and cylindrospermopsins. Microcystins were detected in situ in water samples from four samples E06, E11, E22 and E25. Anatoxins were detected by ELISA in six water samples (E02, E06, E08, E11, E15 and E22), while cylindrospermopsins were not detected in any of the samples.

In addition to in situ analysis of cyanobacterial toxins in the water by ELISA, microbial mats were studied in three laboratories using LC-MS/MS. The analysis of 51 mats (21 collected in 2015 and 30 collected in 2017) conducted in the laboratory of Biogeochemistry and Environmental Conservation (University of Warsaw) revealed the presence of microcystin congeners in only two samples (from 2017); dmMC-LR (*m/z* 981.5) in sample E08, and [ADMAdda^5^]MC-RR (*m/z* 533.8 [M + 2H]^2+^) and [Asp^3^, ADMAdda^5^]MC-LR (*m/z* 1009.5) in sample E30. The analysis conducted in the Division of Marine Biotechnology (University of Gdańsk) detected dmMC-LR (*m/z* 981.5) in sample E08 (Supplementary Figure S2), whilst sample E30 was not analyzed by this laboratory. Sample E11 showed the presence of a peptide with *m/z* 911 and peaks in the fragmentation spectrum characteristic of Adda (*m/z* 135) and Glu-Mdhb (*m/z* 227), but its structure was not able to be elucidated at this point. Nodularin-R, cylindrospermopsin (CYN) and anatoxin-a (ATX) were not detected in any of the samples analyzed at the University of Gdansk (Supplementary Table S3).

Additionally, 16 microbial mats where *mcy* genes were detected by PCR or microcystins were detected by ELISA were cross-checked in the laboratory of Cawthron Institute (New Zealand) using a precursor ion scanning method. This MS/MS technique fragments ions from a relevant mass range (*m/z* 450–1150 to include both doubly- and singly-protonated microcystins) and the precursor ions that produce relevant fragment ions (*m/z* 135 for Adda-containing microcystins and *m/z* 265 for ADMAdda-containing microcystins) are registered. Microcystin precursor ion scanning analysis of the benthic mat samples indicated that 4 of the 16 mat samples (E01, E23, E27 and E30) contained candidate compounds that warranted further investigation. Each of these candidate compounds (15 in total) was analyzed using targeted MS/MS experiments, which revealed that 13 of the candidates were not microcystins. The E30 sample was the only sample, in which microcystins were detected and it contained [Asp^3^, ADMAdda^5^]MC-LR (*m/z* 1009.5) and [Asp^3^, ADMAdda^5^]MC-HarR (*m/z* 533.9 [M + 2H]^2+^ and 1066.8 [M + H]^+^). A fast reaction rate was observed; during micro-scale thiol derivatisation (using β-mercaptoethanol) of the two microcystin congeners from E30, and near-complete conversion was achieved within 2 h (at 30 °C). This observation (in conjunction with the targeted MS/MS analysis) is indicative of Mdha^7^ being present in the microcystins, rather than Dhb^7^.

In the microbial mats analyzed by Cawthron Institute, no anatoxins or cylindrospermopsins; ATX, homoanatoxin-a (HTX), dihydroanatoxin-a (dhATX), dihydrohomoanatoxin-a (dhHTX) CYN and deoxy-CYN (doCYN), were detected by LC-MS/MS MRM (Supplementary Table S3).

## 3. Discussion

In this study we report limited toxin-production by cyanobacteria in microbial mat communities from a cold high-mountain desert (Eastern Pamir, Tajikistan). In contrast to the previous study on benthic communities in Pamir [28], the samples in this study (collected in 2015 and 2017) showed high microbial diversity in the mats sampled with representation from Synechococcales-dominated mats, which were multilayered with variable taxonomic composition, and some *Nostoc*-dominated mats. Despite the high diversity of cyanobacteria in the microbial mats, and occasionally high biomass, we identified only six microcystin congeners in two samples, and in one sample, a potentially new microcystin or nodularin variant was detected. The communities in which microcystin congeners were identified had different a morphology and taxonomic composition, and grew in waterbodies with contrasting physical and chemical parameters. The first sampling site (E08) was a thermokarst pool with a thick “jelly-like” mat, while the second one (E30) was in the form of wrinkled *Nostoc* mat growing in a drying stream at a top of the mountain. The third community (E11) thrived in a thermokarst pool and formed thick multilayer soft mat.

The results of in situ ELISA detection of cyanotoxins were generally not comparable with the LC-MS/MS results and with the PCR amplified microcystin-encoding genes. Only in one case (sample E11), *mcyE* was detected with HEP primers and microcystins by ELISA and by LC-MS/MS. In this sample, the presence of a peptide with *m/z* 911 and fragment ions characteristic for microcystins/nodularins were detected, but we were unable to elucidate its structure. There are plans to conduct further analysis on this mat in the future. No anatoxin-encoding genes were detected, despite positive results in six samples using the anatoxin-a ELISA. Anatoxins (including other anatoxin congeners besides solely ATX; HTX, dhATX and dhHTX) were also not detected by LC-MS/MS, reinforcing the results of the molecular analysis. Other authors have also reported frequent false positive results of microcystin-based ELISAs [33,34]. We encountered additional obstacles here, such as high salinity in some of the samples, which could have influenced the analyses, giving false positive results for both the microcystin and possibly the ELISAs that were conducted to test for anatoxins. Interference from high salinity has been suggested in the Abraxis protocol for the anatoxin ELISA (Warminster, PA, USA). No cylindrospermopsins were detected in situ from the overlaying water using ELISA, or in the mat samples using LC-MS/MS.

The comparative analysis of the taxonomic composition of communities based on phylogenetic placement of V3-V4 16S rRNA gene sequences and screening of genes-encoding toxins production revealed some potential toxin producers. The results of the analyses acquired for sample E30, in which we identified the microcystin congeners [Asp^3^, ADMAdda^5^] MC-LR and [Asp^3^, ADMAdda^5^] MC-HarR, indicated that it was probably produced by *Nostoc* sp. We identified *Nostoc* sp. in the sample based on morphology and V3-V4 amplicons as well as by phylogenetic analysis of *mcyE* gene, which placed the obtained sequence, together with two other sequences from this study, in a sister clade to *Nostoc*-*Nodularia* clade. In previous studies on microcystin-producing *Nostoc* from Arctic and Antarctic environments [3,21,35], the presence of the ADMAdda^5^ modification has frequently been accompanied by the Dhb^7^ modification (or the precursor amino acid, Thr^7^). During the present study, an interesting observation was the presence of the ADMAdda^5^ modification in conjunction with the Mdha^7^ conventionally observed in microcystins, especially those produced by planktonic cyanobacteria.

Because of limited availability in the databases of *mcyE* sequences of cyanobacteria from identified species, we have often obtained a high similarity of identified sequences to those named as “uncultured bacteria clone”, or a 92% similarity with *Planktothrix rubescens,* though this species has not been identified in this particular community. However, in sample E08 with toxin-encoding genes (*mcyD, mcyE*, HEP amplified *mcyE*) and microcystins (dmMC-LR), no *Planktothrix* sp. was identified beside *Phormidium* CYN64, which after verification using raxml-HPC method proved to be *Leptolyngbya* CYN68. This sequence, according to the GenBank database (BLAST), was highly similar to *Timaviella* sp. from Synechococcales. The high similarity and 100% of coverage of V3-V4 sequences, the raxml-HPC method, as well as the results of the BLAST identification showed that these sequences belong to a cyanobacterium from Synechococcales.

Comparing the composition of benthic communities from the Svalbard archipelago, where Oscillatoriales and Nostocales dominated [21], Pamirian microbial mats consist mainly of Synechococcales that are less studied and not as rich as other orders in potentially toxigenic taxa. However, there are reports indicating the presence of HEP-amplified genes (*mcyE* and/or *ndaF*) in planktonic *Leptolyngbya* sp. [36] or *mcyE* gene in *Synechococcus* sp. [37]. Dixit et al. [38] provided information on the detection by ELISA of microcystins produced by *Oscillatoria* sp. RBD01 and *Leptolyngbya* sp. RBD05 in the Ganga River. Other records concern sponge-associated Synechococcales taxa of *Leptothoe* spp. (*Le. s*i*oniana*, *Le. spongobia*, *Le. kymatousa*), which were identified as MC-RR producers, although microcystin production was not confirmed under laboratory conditions [39].

Our study revealed not only limited production of microcystins, but also low representation of toxin-encoding genes in the samples. The genetic analyses of microbial communities from extreme environments suffer from many biases and depend on biogeographic differences in taxonomic composition and structure and presence of different metabolites and ions, which could inhibit the PCR and DNA extraction. Additionally, most primers available in the literature were designed for planktonic cyanobacteria, which may partly explain the difficulties of obtaining the PCR amplicons from toxigenic species in microbial mats. The HEP primers [40] only ideally fit *mcyE* sequences from *Microcystis*, while *mcyE* primers [41] additionally ideally fit the *Fischerella* sequences. The uncertainty about primers’ universality to detect toxin-encoding genes in planktonic taxa is discussed in Bukowska et al. [24].

Another interesting phenomenon is potential cyanotoxicity of the species from order Synechococcales, which as stated above is poorly studied. We could not determine whether *Leptolyngbya* sp. or a closely related species from Synechococcales is a new putative toxin-producing cyanobacterium, though sequences of this cyanobacterium dominated in two samples in which the *mcyD* and HEP amplicons were detected. Moreover, the identical *mcyE* gene sequence was amplified from the mat sample E08 and from the single-filament PCR of the culture isolated from this microbial mat (E8 culture hep2), in which *Leptolyngbya* sp. and *Nostoc* sp. were present, but no *Planktothrix* was detected. The *mcyE* gene sequences from E8 culture hep2 and E08 were nested in the *Planktothrix* clade on the phylogenetic tree of *mcyE* gene sequences (Figure 5), strongly indicating that these genes are derived from *Planktothrix*. Interestingly, Bukowska et al. [37] obtained *mcyE* gene sequences from a *Synechococcus* culture which were almost identical to sequences from *Planktothrix*. Similarly, Frazão et al. [36] sequenced *mcyE* genes from a culture of *Leptolyngbya* (LEGE 06010), which were highly similar to *Microcystis*. The authors suggested the possibility that *Leptolyngbya* might have obtained microcystin-encoding genes by horizontal gene transfer. Whether *mcyE* genes from sample E08 are coming from an undetected *Planktothrix* species or from a *Leptolyngbya* species which obtained the *mcyE* gene from *Planktothrix* needs further detailed studies.

A question arises, however, as to why no microcystins were detected in the other samples, in which we identified *Nostoc* sp. or *Leptolyngbya* sp. Results from various aquatic environments suggest that toxigenic cyanobacteria and cyanotoxins are common in waterbodies. Kobos et al. [42], studying freshwater lakes in Poland, found cyanotoxins in 24% of analyzed samples by HPLC and in 73% by LC-MS/MS. Mantzouki et al. [43], analyzing plankton samples from 137 lakes across Europe in European Multi Lake Survey (EMLS), found microcystin variants in 93% of the studied EMLS lakes, with MC-YR being the most common congener. Although these lakes are thought to represent wide trophic status range, they could have been slightly biased towards more productive ecosystems, because most of the lakes in Europe are presently eutrophic [9]. Waterbodies sampled in this study were characterized by low total phosphorus and nitrogen concentrations. However, the low nutrient content in water cannot easily explain the growth of microbial mats and low toxin production by benthic cyanobacteria, because they can acquire nutrients from the water column and from sediments as discussed in Wood et al. [19]. As summarized by Kleinteich et al. [21] between 20% and 96% of studied samples of Arctic and Antarctic benthic communities contained cyanotoxins, mostly microcystin congeners. Records from Asia—not distinguishing between benthic and planktonic communities—revealed that microcystins were detected as the most abundant toxins in freshwater habitats (79%; 132 out of 168) [25]. These were produced by taxa from the orders Chroococcales (*Microcystis* spp.—162 records), Nostocales (*Anabaena* spp.—79, *Nostoc* spp.—30), Oscillatoriales (*Oscillatoria* spp.—52) and Synechococcales (*Aphanocapsa* spp.—33) [25]. Taking this all into consideration, the low production of cyanotoxins in Pamirian mats revealed by our study was unexpected. It might be because cyanobacteria occurring in these environments were very different from those identified in other environments, and because in this study, we have detected only the similar microcystin-producers. Kleinteich et al. [21], in their studies of polar environments, demonstrated a high diversity of poorly studied structural congeners of microcystins which were difficult to identify, similar to the peptides we observed in sample E11. The presence of new congeners can be overlooked when targeted methods are used; hence, we used a range of toxin detection strategies during our study.

Another possible explanation for the low-level detection of toxigenic taxa and toxins in the Pamir mat samples is that in this extreme environment, the costly production of cyanotoxins is not a trait which is useful. As it was stated by Holland and Kinnear [11] cyanotoxins production plays multiple roles for cyanobacteria but it requires high-energy input. The very short vegetation period, high UV and visible light insolation, variable temperature seasonally and daily and often high salinity may exert a lot of stress on cyanobacteria, preventing them from committing additional energetic costs on toxin production. Further studies of the whole metagenomes of investigated microbial mats from Pamir should be undertaken to identify if; (1) full toxin-encoding gene operons are present in studied samples but the cyanobacteria do not produce enough toxins to be detected, or (2) whether the species have lost portions of the microcystin gene operons and thus are not able to produce toxins, or (3) if toxigenic taxa are mostly absent in the communities, potentially being eliminated by evolutionary forces.

Although the common anatoxin-producers; *Microcoleus* and *Phormidium* [10,20,44,45], were abundant in samples V3–V4, we did not detect genes encoding anatoxin production. However, failure to detect *anaC* genes, as well as anatoxins in all samples suggests that anatoxin-producing cyanobacteria may be even less common in this environment than those producing microcystins. Furthermore, there was little confirmation of saxitoxins in this environment as *sxtA* genes were amplified only in one sample and we did not test for saxitoxins using chemical or biochemical methods during this study. Cylindrospermopsins were not detected in water samples screened by ELISA, nor in microbial mat samples by LC-MS/MS. Further studies on the occurrence of these toxins in microbial mats in Pamir’s cold mountain deserts are needed.

Microbial mats occurring in various waterbodies in cold high mountain desert in Eastern Pamir are sources of diverse cyanobacterial taxa, including new potential toxin producers. Based on the *mcyE* gene phylogeny, three considerably distantly related genes, closely related to *mcyE* gene from *Nostoc* sp., Oscillatoriales (*Planktothrix* and *Oscillatoria*) and the uncultured cyanobacterium were present in these communities. We did not detect a high diversity of toxins in the analyzed samples. The microcystin congeners [Asp^3^, ADMAdda^5^]MC-LR and [Asp^3^, ADMAdda^5^]MC-HarR, were presumably produced by Nostocales (*Nostoc* sp.). We have not identified a potential producer of the dmMC-LR observed, although some analyses suggest that a species belonging to Synechococcales might be a putative producer, which carried *mcy* genes that grouped with Oscillatoriales. These data suggest that microcystin, anatoxin and cylindrospermopsin production by cyanobacteria in the cold high mountain desert environments of Eastern Pamir (Tajikistan) is not particularly prevalent.

## 4. Materials and Methods

### 4.1. Study Area, Sampling and Sampling Sites

The area investigated is located in the eastern part of the Pamir Mountains in Tajikistan. The samples were collected from the pools, ponds, small water bodies and thermokarsts surrounding nine Pamirian lakes, including Bulunkul (A03, E01, E02, E03), Sassykkul (A04-A09, E04-E08), Khargush (E12), Chukurkul (A10-A11, E13), Yashilkul (A02, E14), Rangkul (A15-A21, E17-E19), Shorkul (E20-E22), Zorkul (E23-E24), Karakul (E25-E30), as well as streams and pools near Alichur village (E09-E11, E15-E16). Twenty-one samples were collected in July 2015 (A01-A21) and thirty samples (E01-E30) were collected in July 2017. The study area in Eastern Pamir is shown on the map (Figure 2) and localization of sampling places is summarized in Supplementary Table S1. Each sample was divided into three subsamples, one of which was fixed with DESS solution and the others were dried and stored in sterile Petri dishes. The subsamples fixed in DESS were stored in the fridge, part of the dried samples served as inoculum to obtain cyanobacterial cultures, as well as for DNA extraction, while the remainder of the lyophilized samples were individually powdered for LC-MS/MS analysis.

### 4.2. Physical and Chemical Parameters of Water in Studied Reservoirs

The physical and chemical water quality parameters—electrical conductivity (EC), temperature (T) and pH—were measured in situ using a HachHQ40d portable multimeter. The rest of the parameters were studied in the laboratory of Biogeochemistry and Environmental Conservation (University of Warsaw) and measured with a Jenway PFP7 flame photometer. The estimation of organic carbon was carried out using multi n/c 3100 Analytik Jena AG and total nitrogen was evaluated according manufacture protocol. Ammonium concentration was conducted based on Nessler method (8038 method), iron was evaluated using 8008 method (with 1,10 phenanthroline indicator) and phosphate using the molybdenum method (method 8048). The results of the physical and chemical analyses of samples from 2017 can be found in Supplementary Table S2.

### 4.3. DNA Extraction, Genes Amplification, Sequencing and Sequences Processing

Genomic DNA was extracted from homogenized dry mats as well as samples fixed with DESS solution using both E.Z.N.A.^®^ Soil DNA Kit (Omega Bio-tek, Norcross, GA, USA and Soil DNA Purification Kit (GeneMATRIX, EURx Ltd., Gdańsk, Poland. Extractions were performed according to manufacture protocols. The V3-V4 hypervariable region of the 16S rRNA gene was amplified using universal primers [46]. The first PCR has been performed using HotStarTaq DNA Polymerase (Qiagen, Hilden, Germany. The total volume of reaction mix containing (25 µL) 2.5 µL PCR Buffer, 5 µL Q-solution, 0.3 µL dNTP, 0.1 mL Taq, 0.5 µM of each primer, 15.1 µL nuclease-free water and 1 µL (5–65 ng) of DNA template was prepared. The amplification was conducted with thefollowing conditions: 15 min of denaturation at 95 °C, followed by 25 cycles of 95 °C for 30 s, 55 °C for 30 s, 72 °C for 30 s and a final elongation at 72 °C for 10 min. Amplicon sequencing was performed in the Science and Technology Park “Bionanopark” (Łódź, Poland) using Illumina MiSeq platform and V3 MiSeq reagent kit. The targeted sequencing depth was 150,000 per sample. The quality of the sequences was checked using “demux” plugin. The demultiplexed reads (2 × 300 bp) were processed in QIIME2 environment using version 2019.07 [47]. The paired sequences were trimmed and dechimerized using the “DADA2” [48] plugin in QIIME2. Clustering of the sequences was carried out based on ASVs taxonomic assignment was performed using SILVA-based classifier (“silva-132-99-nb-classifier”). The scripts used for analyses of sequences are available in Supplementary Table S4. The sequences were submitted to BioProject database with ID SUB7044730.

Phylogenetic placement of cyanobacterial ASVs was conducted to verify taxonomic position of the most abundant taxa as well as potential toxins producers. The query sequences were aligned to reference sequences from the Cydrasil database using the PaPaRa alignment algorithm and raxml-HPC method [49]. The sequences were mapped on Cydrasil-ML-tree-bs 1000 [49] and the nomenclature used is maintained by Guiry and Guiry [50].

Amplification of the genes encoding microcystin (*mcyD* and *mcyE*), and microcystin and nodularin (*mcyE* and/or *ndaF*), saxitoxin (*sxtA*) and anatoxin (*anaC*) was conducted by classical PCR using universal primers (Table 1). The PCR mix consisted of 1 µL (4–10 ng/ µL) of DNA template, 1.25 µL of each forward and reverse primers, 12.5 µL of FastGene^®^ Optima HotStart ReadyMix (Nippon Genetics Co., Ltd., Tokyo, Japan) and 9 µL of water. The cycling conditions were as following: 3 min initial denaturation at 95 °C, followed by 30 cycles of 95 °C for 15 s, 44–58°C for 30 s, and 72 °C for 1 min, and a final elongation at 72 °C for 10 min. The conditions were optimized for investigated DNA template. The PCR products were stained with DNA dye and bands excised from the gel and purified. PCR products were at the Genomed join-stock company (Warsaw, Poland). The PCRs of investigated regions were repeated a few times for each sample and then sequenced. The best quality sequences were used for phylogenetic analyses and marked on phylogenetic tree as for example: E23 hep or E19 hep2 depending on the amplification.

Phylogenetic analysis of *mcyE* genes amplified with HEP primers. Sequences obtained from PCR reactions were paired using SeqMan (DNASTAR software, Lasergene, version 15, Madison, WI, USA manually trimmed and were used as queries for Blast searches in GenBank (http://blast.ncbi.nlm.nih.gov/Blast.cgi). One hundred sequences with the lowest E-value were pooled for each sequence. All collected sequences were aligned using Muscle [51], implemented in Mega 7 [52], and redundant sequences were removed from the alignment. The *mcyE* DNA sequences were translated into protein and aligned by Muscle. The Maximum Likelihood analysis was performed and GTR model parameters were estimated with the PHYML program [53]. Tree stability was estimated by the aLRT test [54].

### 4.4. In Situ Cyanotoxins Detection

The presence of microcystins, cylindrospermopsins and anatoxins was analyzed using ELISA strip tests (Abraxis, Warminster, PA, USA—presently Eurofins). The ELISA strip tests were performed on the surface water collected above the benthic mats. The analyses were completed according to manufacturer’s instructions. The microcystins strip test detection limit was 0.5 ng/mL and it assessed concentrations of microcystins between 0 and 5 ng/mL. The anatoxins (VFDF) strip test sensitivity was 0.4 ng/mL and it estimated concentrations of ATX in water up to 2.5 ng/mL. The cylindrospermopsins strip test assessed the concentrations of CYN between 0 and 10 ng/mL with a detection limit of 0.5 ng/mL. According to manufactures’ instructions, the strip tests results give the qualitative information of whether the cyanotoxins were detected by ELISA analyses within the given range or not.

### 4.5. Extraction and Toxin Analysis using LC-MS/MS

Microcystin analysis by MRM LC-MS/MS (performed in the laboratory of Biogeochemistry and Environmental Conservation, University of Warsaw, Poland; and the Division of Marine Biotechnology, University of Gdańsk, Gdańsk, Poland). In the laboratory of Biogeochemistry and Environmental Conservation, University of Warsaw, all 51 samples were analyzed, while in the laboratory of the Division of Marine Biotechnology, University of Gdańsk, 12 samples collected in 2017 were cross-checked. The 12 lyophilized mats (5 mg) were homogenized and extracted in 2 mL 75% methanol by vortexing for 3 min and sonication in ultrasonic bath for 1 min. After centrifugation for 10 min at 14,000× *g*, the samples were transferred to chromatographic vials and analyzed with LC-MS/MS system. Chromatographic separation of sample components was performed on Zorbax Eclipse XDB-C18 column (4.6 mm ID (inner diameter) × 150 mm, 5 µm; Agilent Technologies, Santa Clara, CA, USA) by gradient elution with a mixture of 5% acetonitrile in Milli-Q water (solvent A) and acetonitrile (solvent B), both containing 0.1% formic acid. The system was composed of an Agilent 1200 (Agilent Technologies, Waldboronn, Germany) chromatograph coupled online to a hybrid triple quadrupole/linear ion trap mass spectrometer (QTRAP5500, Applied Biosystems, Sciex, Concord, ON, Canada). Mass spectrometer with a turbo ion source (550 °C, 5.5 kV) operated in positive mode. For non-target analyses the information-dependent acquisition method (IDA) was used. For ions within the m/z range 500–1250 and signal intensity above the threshold of 500,000 cps, fragmentation spectra at a collision energy of 60 eV and declustering potential of 80 eV were acquired. In addition, to detect cyanotoxins (microcystins, nodularin, anatoxin-a and cylindrospermopsin) that could be present at trace amounts, and for the quantitative analysis of the compounds in positive samples, MRM with the following transitions was used: MC-LR: 995 → 375 (CE 65), 135 (CE 93), 107 (CE 121), 213 (CE 79); dmMC-LR: 981 → 135 (CE 60), 375 (CE 60), 213 (CE 60); MC-LA: 910 → 135 (CE 79), 213 (CE 79), 375 (CE 53); MC-LY: 1002 → 375 (CE 75), 135 (CE 77); MC-LW: 1025 → 135 (CE 85), 107 (CE 111), 375 (CE 53), 213 (CE 67); MC-RR: 519 → 213 (CE 47), 329 (CE 41), 135 (CE 39), 107 (CE 65); MC-LF: 986 → 135 (CE 77), 213 (CE 79); MC-YR: 1045 → 213 (CE 75), 135 (CE 127), 375 (CE 69); nodularin-R: 825→135 (CE 80), 227 (CE 65), 163 (CE 60); ATX: 166→149 (25 CE), 131 (25 CE), 107 (25 CE), 91 (25 CE); CYN: 416→336 (47 CE), 274 (50 CE), 194 (40 CE), 176 (47 CE). The presence of microcystins was also confirmed using enhanced product ion mode (CE 60 ± 20). Standards of microcystins used in the analysis were from Alexis Biochemicals. The detection limits for used standards was 0.1 ng/mL. For data acquisition and processing Analyst^®^ Software (version 1.5.1, Applied Biosystems, Concord, ON, Canada) was used.

Microcystin analysis by precursor ion scanning LC-MS/MS (carried out at Cawthron Institute, Nelson, New Zealand) was carried out on 16 samples collected in 2017. The freeze-dried cyanobacteria mats were homogenized with a sterile spatula, weighed into a microcentrifuge tube (23–67 mg) and extracted in 80% aqueous methanol (v/v) with 0.1% formic acid (v/v; 1 mL). The samples were sonicated for 1 h in a bath sonicator cooled with ice, clarified by centrifugation (10,000× *g*, 5 min) and the extract was transferred to a glass vial for LC-MS/MS analysis. Microcystin extracts were analyzed using the precursor ion scanning method described in Kleinteich et al. [21] using an Acquity I-Class ultra-performance liquid chromatography system (UPLC; Waters, Milford, MA) coupled to a Xevo-TQS triple quadrupole mass spectrometer (Waters, Manchester, UK). Compound separation was achieved using an Acquity BEH-C18 UPLC column (Waters Micromass; 1.7 µm, 50 × 2.1 mm) and the chromatography conditions described in Kleinteich et al. [21].

The candidate microcystins identified using the precursor ion scanning method were further investigated by generating MS/MS spectra for each ion of interest. Tandem MS spectra were collected in positive ion mode over an *m/z* range of 100–1200. Compounds that were presumed to be microcystins containing two arginine residues (e.g., MC-RR) were fragmented using a collision energy of 25–30 V and compounds presumed to be microcystins containing one or no arginine residues (e.g., MC-LR or MC-LA respectively) were fragmented using a collision energy of 40–45 V. The spectra were primarily annotated with the assistance of the software mMass. When discrepancies were apparent, the spectra were annotated manually using previously published MS/MS investigations of microcystins as a guide. To confirm whether Mdha or Dhb were present in the microcystins identified, a micro-scale thiol derivatization was performed. Microcystin solutions were reacted with β-mercaptoethanol as described in Puddick et al. [10], but using the precursor ion scan method. If no reaction had occurred within 2h at 30 °C, the microcystin was classified as containing Dhb^7^. A control reaction containing MC-RR, MC-LR and MC-LA was run in parallel to confirm the reaction rate for Mdha.

Anatoxin and cylindrospermopsin analysis by MRM LC-MS/MS (performed at Cawthron Institute, New Zealand) was also performed on 16 samples. Freeze-dried cyanobacteria mats were homogenized, weighed into a microcentrifuge tube (95–108 mg) and extracted in 0.1% formic acid in Milli-Q water (v/v; 1 mL). The samples were frozen, then thawed in bath sonicator for 30 min. The freezing and thawing process was repeated two more times, before clarification by centrifugation (10,000× *g*, 5 min) and the extract was transferred to a glass vial for LC-MS/MS analysis. Toxin extracts were analyzed using the MRM method described Wood et al. [44] using an Acquity I-Class UPLC system coupled to a Xevo-TQS triple quadrupole mass spectrometer and a Thermo Hypersil Gold aq. column (1.9-μm; 50 × 2.1 mm). The following MRM transitions were used: ATX: 166.1 → 149 (CE 13), 131 (CE 17); HTX-: 180.1 → 163 (CE 13), 91 (CE 17); dhATX: 168.1 → 56 (CE 20); dhHTX: 182.1 → 57 (CE 20), 98 (CE 20); CYN: 416.3 → 194.15 (CE 35), 336.3 (CE 22); doCYN: 400.3 → 194.15 (CE 35), 320.3 (CE 22). External standards were used for calibration, using dilutions of certified reference materials for ATX and CYN (National Research Council; Ottawa, Canada) and an in-house standard for dhATX standardized by quantitative nuclear magnetic resonance spectroscopy. The ATX standard was used to quantify ATX and HTX, the dhATX standard was used to quantify dhATX and dhHTX, and the CYN standard was used to quantify CYN and doCYN. All calibrations used a relative response factor of 1, acknowledging that the response ratio between ATX / HTX, dhATX / dhHTX and CYN / doCYN was not confirmed experimentally (due to a lack of reference material). The limit of detection was 0.1 ng/mL for ATX and dhATX, and 0.3 ng/mL for CYN. A linear response was observed for all standards up to at least 18 ng/mL.

## Figures and Tables

**Table 1:** Primers and PCR annealing temperatures used in this study to investigate the presence of genes involved in cyanotoxin production.

Target Gene	Primer	Sequnces 5′ to 3′	AT*	References
***mcyE*, *ndaF***	HEPf	TTTGGGGTTAACTTTTTTGGGCATAGTC	56	[40]
	HEPr	AATTCTTGAGGCTGTAAATCGGGTTT		
***mcyA***	mcyA-Cd1F	AAAATTAAAAGCCGTATCAAA	45	[55]
	mcyA-Cd1R	AAAAGTGTTTTATTAGCGGCTCAT		
	mcyD F2	GGTTCGCCTGGTCAAAGTAA		
***mcyD***	mcyD R2	CCTCGCTAAAGAAGGGTTGA	46	[56]
***mcyE***	mcyE-F2 mcyE-R4	GAAATTTGTGTAGAAGGTGC AATTCTAAAGCCCAAAGACG	56	[41]
	anaC-gen F	TCTGGTATTCAGTCCCCTCTAT	58	[57]
***anaC***	anaC-gen R	CCCAATAGCCTGTCATCAA		
	sxtSUL-F	ATTTGTTGTTGGTGCTCCTA	44	[58]
***sxtA***	sxtSUL-R	TTAATCGCAGTTCAGGGTC		

* AT = Annealing Temperature (°C).

## Supplementary Materials

The following are available online at https://www.mdpi.com/2072-6651/12/4/244/s1: Table S1. Geographic localization of the sampling sites; Table S2. Environmental characteristics of the studied water reservoirs; Table S3. Distribution of *mcy, nda, ana* and *sxt* genes as well as studied toxins using ELISA and LC-MS/MS methods. Table S4. Scripts used for analyses of sequence data in QIIME2 (version 2019.4); Figure S1. Phylogenetic tree with mapped V3-V4 16S rRNA sequences of cyanobacteria from microbial mats; Figure S2. Fragmentation spectrum of microcystin dmMC-LR detected in sample E08 (2Cyx8) with characteristic product ions. The phylogenetic tree is available under the following link https://itol.embl.de/tree/21287138665131573044368# Login: nkhomutovska, password: rGDlKYQa.
