# Supplementary material for: Limited Microcystin, Anatoxin and Cylindrospermopsin Production by Cyanobacteria from Microbial Mats in Cold Deserts
Source: Toxins (Basel). 2020 Apr 11;12(4):244. doi: 10.3390/toxins12040244 (PMC7232524; doi:10.3390/toxins12040244)
Supplement: Supplementary file 1 [file toxins-12-00244-s001.pdf]

Supplementary Materials: Limited Microcystin, Anatoxin and Cylindrospermopsin Production by Cyanobacteria from Microbial Mats in Cold Deserts

Nataliia Khomutovska, Małgorzata Sandzewicz, Łukasz Łach, Małgorzata Suska-Malawska, Monika Chmielewska, Hanna Mazur-Marzec, Marta Cegłowska, Toirbek Niyatbekov, Susanna A. Wood, Jonathan Puddick, Jan Kwiatowski and Iwona Jasser

Table S1. Geographic localization of the sampling sites.

Sample ID	Localization	Reservior type	Mat type	Latitude, Longitude, Altitude**
A01	Khorog-Langar	Stream	*Phormidium* type	N37°29.57.4', E071°32.92.1', 2380
A02	Yashilkul	Stream	*Phormidium* type	N37°43.937', E072°53.480', 3754
A03	Bulunkul	Thermokarst p.*	Multilayer soft	N37°43.937', E072°53.48.0', 3752
A04	Sassykkul	Pool	Epiphytes	N37°42.273', E072°56.711', 3830
A05	Sassykkul	Pool	Multilayer hard	N37°40.708', E073°10.873', 3830
A06	Sassykkul	Pool	*Phormidium* type	N37°40.690', E073°10.917', 3834
A07	Sassykkul	Pool	Multilayer hard	N37°40.679', E073°10.882', 3837
A08	Sassykkul	Stream	*Phormidium* type	N37°40.671', E073°10.882', 3838
A09	Sassykkul	Lake shore	*Phormidium* type	N37°26.558', E073°04.792', 3993
A10	Chukurkul	Lake shore	Amorphous	N37°33.120', E073°06.567', 3966
A11	Chukurkul	Pool	*Phormidium* type	N37°33.120', E073°06.567', 3966
A12	Yashilkul	Pool	*Phormidium* type	N37°46.584', E072°44.32.4', 3710
A13	Yashilkul	Pool	*Phormidium* leather-like	N37°43.585', E072°44.30.8', 3697.
A15	Rangkul	Stream	*Phormidium* type	N38°28.319', E074°16.83,9', 3780
A16	Rangkul	Pool	Multilayer soft	N38°28.429', E074°16.52.3', 3783
A17	Rangkul	Pool	*Phormidium* type	N38°28.428', E074°16.52.3', 3800
A18	Rangkul	Pool	*Phormidium* type	N38°28.493', E074°16.56.6', 3817
A19	Rangkul	Pool	Multilayer soft	N38°28.886', E074°16.75.3', 3830
A20	Rangkul	Pool	*Phormidium* type	N38°28.164', E074°16.77.7', 3760
A21	Rangkul	Pool	Multilayer hard	N38°28.164', E074°16.77.7', 3760
E01	Bulunkul	Pool	Jelly-like	N37°42.355', E072°58.367', 3712
E02	Bulunkul	Thermokarst p.*	Multilayer soft	N37°42.358', E072°58.364', 3711
E03	Bulunkul	Thermokarst p.*	Multilayer soft	N37°42.360', E072°58.357', 3713
E04	Sassykkul	Lake shore	*Phormidium* beneath soil	N37°40.699', E073°10.487', 3796
E05	Sassykkul	Thermokarst p.*	Jelly-like	N37°40.689', E073°10.517', 3795
E06	Sassykkul	Thermokarst p.*	Multilayer soft	N37°40.662', E073°10.645', 3795
E07	Sassykkul	Pool	Multilayer soft	N37°40.621', E073°10.686', 3798
E08	Sassykkul	Thermokarst p.*	Jelly like	N37°40.669', E073°10.670, 3793
E09	Alichur	Pool	*Phormidium* beneath soil	N37°43.682', E073°08.536', 3777
E10	Alichur	Thermokarst p.*	Multilayer hard	N37°43.863', E073°08.209', 3775
E11	Alichur	Thermokarst p.*	Multilayer soft	N37°43.905', E073°08.209', 3762
E12	Khargush	Stream	*Phormidium* type	N37°28.452', E073°04.481', 4241
E13	Chukurkul	Lake shore	Amorphous	N37°33.136', E073°06.608', 3917
E14	Yashilkul	Stream	*Phormidium* type	N37°43.879', E072°53.428', 3751
E15	Alichur	Stream	*Phormidium* type	N37°46.027', E073°01.195, 3703

E16	Alichur	Stream	*Phormidium* type	N37°44.552', E073°03.803', 3742
E17	Rangkul	Thermokarst p.*	Multilayer hard	N38°28.441', E074°16.208', 3758
E18	Rangkul	Pool	Multilayer soft	N38°28.393', E074°15.970, 3759
E19	Rangkul	Pool	*Phormidium* beneath soil	N38°28.264', E074°15.817', 3757
E20	Shorkul	Pool	*Phormidium* beneath soil	N38°26.786', E074°10.120', 3750
E21	Shorkul	Pool	Multilayer soft	N38°26.956', E074°10.428', 3751
E22	Shorkul	Thermokarst p.*	Multilayer soft	N38°28.569', E074°16.314', 3756
E23	Zorkul	Lake shore	Amorphous	N37°27.41525, E73°40.4970', 3989
E24	Zorkul	Lake shore	*Phormidium* type	N37°27.41525, E73°40.4970', 3989
E25	Karakul	Pool	*Nostoc* type	N39°01.241', E073°33.282', 3891
E26	Karakul	Pool	*Phormidium* beneath soil	N39°01.369', E073°33.271', 3885
E27	Karakul	Pool	Multilayer soft	N39°01.369', E073°33.271', 3885
E28	Karakul	Pool	*Nostoc* type	N39°01.951', E073°33.462', 3887
E29	Karakul	Stream	*Phormidium* type	N38°51.354', E073°24.525', 4521
E30	Karakul	Stream	*Nostoc* type	N38°50.708', E073°23.660', 5006

*Thermokarst p.—Thermokarst pool, **m a.s.l. (metres above sea level).

Table S2. Environmental characteristics of the studied water reservoirs.

Sample ID	T	pH	EC	C org	N tot	P tot	PO_4	NH_3	NO_2/NO_3
E01	24.7	8.7	600	14.1	0.99	0.17	NA	NA	NA
E02	18.5	7.1	475	11.2	0.74	0.03	0.010	0.080	0.215
E03	18.5	7.5	673	13.5	0.89	0.02	0.001	0.038	0.008
E04	17.5	9.9	79500	37.1	19.04	0	2.499	0.035	1.393
E05	11.4	6.9	366	7.1	0.55	0.02	0.008	0.155	0.017
E06	16	7.1	355	6.9	0.45	0.10	0.008	0.148	0.143
E07	17.4	9.9	26800	186.9	14.93	0.69	0.083	0.654	0.316
E08	14	7.1	347	7.5	0.52	0.02	0.005	0.038	0.102
E09	22	8.9	2720	47.9	3.62	0.05	0.006	0.103	0.011
E10	13.7	7.5	267	3.6	0.27	0.01	0.004	0.072	0.16
E11	15.5	7.2	273	10.5	8.67	0.03	0.005	0.101	0.156
E12	21	9.1	1283	9.3	14.74	0.09	0.004	0.065	0.236
E13	16.6	9.1	58300	55.3	4.98	0	0.038	0.232	0.322
E14	35	NA	NA	1.2	8.13	0.03	0.023	0.035	0.25
E15	47.7	6.8	1791	2.7	13.30	0.01	0.009	0.277	0.237
E16	13.8	6.3	4220	8.4	0.80	0.01	0.012	0.586	0.059
E17	11.3	7.8	711	4.6	0.36	0.01	0.002	0.024	0.018
E18	18.1	7.4	720	5.8	0.70	0.06	0.004	0.154	0.056
E19	19	8.3	1270	15.7	1.34	0.01	0.006	0.095	0.018
E20	NA	NA	NA	33.6	16.73	0.18	0.006	0.085	0.196
E21	23.8	7.9	4200	32.5	2.47	0.09	0.001	0.066	0.007
E22	19.4	7.2	1120	8.7	9.74	0.02	0.004	0.057	0.341
E23	20	7.8	552	24.3	2.74	0.04	0.027	0.139	0.424
E24	NA	NA	NA	13.3	40.79	0	0.003	0.015	29.56
E25	19	8.2	1089	13.4	4.58	0.06	0	0.033	0.029
E26	25.6	8.1	17530	79.4	4.71	0.14	0.003	0.109	0.459
E27	26.9	8.7	430	8.8	12.55	0.05	0.003	0.048	0.02
E28	29.2	8.1	401	13.5	11.61	0.06	0.002	0.125	0.717
E29	10	NA	NA	NA	NA	NA	NA	NA	NA
E30	10	NA	NA	NA	NA	NA	NA	NA	NA

*Ions concentrations in mg L^{-1}, EC in μS cm^{-1}, T (temperature) in °C.

Table S3. Results of distribution of *mcy, nda, ana* and *sxt* genes as well as studied toxins using PCR, ELISA and LC-MS/MS methods.

Sample ID	PCR *mcyA*	PCR *mcyD*	PCR *mcyE*	PCR *mcyE+ndaF*	PCR *anaC*	PCR *sxtA*	ELISA MC	ELISA ATX-a	ELISA CYN	LC-MS/MS MCs	LC-MS/MS ATX-a	LC-MS/MS CYN
E01	0	1	0	1	0	0	NA	NA	NA	0	0	0
E02	0	0	0	0	0	1	0	1	0	0	0	0
E03	0	0	0	0	0	0	NA	NA	NA	0	NA	NA
E04	0	0	1	0	0	0	NA	NA	NA	0	0	0
E05	0	1	0	1	0	0	0	0	0	0	0	0
E06	0	0	0	0	0	0	1	1	0	0	0	0
E07	0	0	0	0	0	0	NA	NA	NA	0	NA	NA
E08	0	1	1	1	0	0	0	1	0	dmMC-LR * ** [1]	0	0
E09	0	0	0	1	0	0	NA	NA	NA	0	0	0
E10	0	1	0	1	0	0	0	0	0	0	0	0
E11	0	0	0	1	0	0	1	1	0	m/z 911 ion with 135 and 227 fragments (MC?)	0	0
E12	0	0	0	0	0	0	NA	NA	NA	0	NA	NA
E13	0	0	0	0	0	0	NA	NA	NA	0	0	0
E14	0	0	0	0	0	0	NA	NA	NA	0	NA	NA
E15	0	0	0	0	0	0	0	1	0	0	0	0
E16	0	0	0	0	0	0	NA	NA	NA	0	NA	NA
E17	0	0	0	0	0	0	NA	NA	NA	0	NA	NA
E18	0	0	0	0	0	0	0	0	0	0	NA	NA
E19	0	1	0	1	0	0	NA	NA	NA	0	0	0
E20	0	0	0	0	0	0	NA	NA	NA	0	NA	NA
E21	0	0	0	0	0	0	NA	NA	NA	0	NA	NA
E22	0	0	0	0	0	0	1	1	0	0	0	0
E23	0	1	0	1	0	0	NA	NA	NA	0	0	0
E24	0	0	0	0	0	0	NA	NA	NA	0	NA	NA
E25	0	0	0	0	0	0	1	0	0	0	0	0
E26	0	0	0	0	0	0	NA	NA	NA	0	NA	NA
E27	0	0	0	1	0	0	NA	NA	NA	0	0	0
E28	0	0	0	0	0	0	NA	NA	NA	0	0	0
E29	0	0	0	0	0	0	NA	NA	NA	0	NA	NA
E30	0	0	0	1	0	0	NA	NA	NA	[ADMAdda^5]MC-RR * [Asp^3, ADMAdda^5]MC-LR* [Asp^3,ADMAdda^5]MC-LR *** [Asp^3,ADMAdda^5]MC-HarR ***	0	0

* MCs congeners detected in the laboratory of Biogeochemistry and Environmental Conservation (University of Warsaw), ** MCs congeners detected in the laboratory of in the Division of Marine Biotechnology (University of Gdansk), *** MCs congeners detected in the laboratory of Cawthron Institute (New Zealand). NA—analyses have not been performed. [1]Fragmentation spectrum of microcystin dmMC-LR detected in sample E08 (2Cyx8) with characteristic product ions is presented in Figure S2.

Table S4. Scripts used for analyses of sequences in QIIME2 (version 2019.4).

*The Illumina run included 150 samples. The samples analyzed in this study (samples E01-E30) were filtered from the dataset.

```
source activate qiime2
# import data
```

qiime tools import.
--type 'SampleData[PairedEndSequencesWithQuality]'
--input-path SekwencjeNGS/
--input-format CasavaOneEightSingleLanePerSampleDirFmt
--output-path demux-paired-end_Seqs-all-2019.qza

visualisation
qiime demux summarize
--i-data demux-paired-end_Seqs-all-2019.qza
--o-visualization demux-paired-end_Seqs-all-2019.qzv

Denoise and dereplicate paired-end sequences. This method denoises paired-end sequences, dereplicates them, and filters chimeras
qiime dada2 denoise-paired
--i-demultiplexed-seqs demux-paired-end__Seqs-all-2019.qza
--p-trim-left-f 10
--p-trim-left-r 10
--p-trunc-len-f 300
--p-trunc-len-r 300
--o-table table_Seqs-all-2019_t10.qza
--o-representative-sequences rep-seqs_Seqs-all-2019_t10.qza
--o-denoising-stats denoising-stats_Seqs-all-2019_t10.qza

Assign taxonomy using Silva 132 99% OTUs (full-length, seven-level taxonomy) classifier with filtering sequences that are with less than 0.9 confidence
qiime feature-classifier classify-sklearn
--i-classifier silva-132-99-nb-classifier.qza
--i-reads rep-seqs_Seqs-all-2019_t10.qza
--p-confidence 0.9
--o-classification taxonomy_Seqs-all-2019_t10_silva_full_0.9.qza

#Filtering of Cyanobacterial sequences
qiime taxa filter-seqs
--i-sequences rep-seqs_Seqs-all-2019_t10.qza
--i- taxonomy taxonomy_Seqs-all-2019_t10_silva_full_0.9.qza
--p-include Cyanobacteria
--o-filtered-sequences rep-seqs_Cyanobacteria_Seqs-all-2019_t10_silva_full_0.9

Edit of taxonomy file
qiime tools export
--input-path taxonomy_Seqs-all-2019_t10_silva_full_0.9.qza
--output-path taxonomy-with-spaces_Seqs-all-2019_t10_silva_full_0.9

Edit of taxonomy file
qiime metadata tabulate
--m-input-file taxonomy-with-spaces_Seqs-all-2019_t10_silva_full_0.9 --o-visualization taxonomy-as-metadata_0.9.qzv

Edit of taxonomy file
qiime metadata tabulate
--m-input-file taxonomy-with-spaces_Seqs-all-2019_t10_silva_full_0.9.qza
--o-visualization taxonomy-as-metadata_0.9.qzv

Edit of taxonomy file
qiime metadata tabulate
--m-input-file taxonomy-with-spaces_Seqs-all-2019_t10_silva_full_0.9/taxonomy.tsv
--o-visualization taxonomy-as-metadata_0.9.qzv

```
# Edit of taxonomy file
qiime tools import
--type 'FeatureData[Taxonomy]'
--input-path taxonomy-as-metadata_0.9/metadata.tsv
--output-path taxonomy-without-spaces_0.9.qza

#Filter Cyanobacterial sequences
qiime taxa filter-seqs
--i-sequences rep-seqs_Seqs-all-2019_t10.qza
--i-taxonomy taxonomy-without-spaces_0.9.qza
--p-include Cyanobacteria
--o-filtered-sequences rep-seqs_Cyanobacteria_Seqs-all-2019_t10_silva_full_0.9

# Assign taxonomy using Silva 132 99% OTUs
qiime feature-classifier classify-sklearn
--i-classifier silva-132-99-nb-classifier.qza
--i-reads rep-seqs_Cyanobacteria_Seqs-all-2019_t10_silva_full_0.9.qza
--o-classification taxonomy_Cyanobacteria_Seqs-all-2019_t10_silva_full_0.9_full.qza

# Merge taxonomy and table with numbers of futures
qiime taxa collapse
--i-table table_Seqs-all-2019_t10.qza
--i-taxonomy taxonomy_Cyanobacteria_Seqs-all-2019_t10_silva_full_0.9_full.qza
--p-level 7
--o-collapsed-table collapsed_Cyanobacteria_Seqs-all-2019_t10_silva_full_0.9_full

#Convert biom file
biom convert
--input-fp feature-table.biom
--o Collapsed_taxonomy_Seqs-all-2019_t10_silva_full_0.9.tsv
--to-tsv

#Visualize result
qiime taxa barplot
--i-table table_Seqs-all-2019_t10.qza
--i-taxonomy taxonomy_Seqs-all-2019_t10_silva_full_0.9.qza
--o-visualization Viz_Seqs-all-2019_t10

#Merge taxonomy and future table
qiime taxa collapse
--i-table table_Seqs-all-2019_t10_E.qza
--i-taxonomy taxonomy_Seqs-all-2019_t10_silva_full_0.9.qza
--p-level 7
--o-collapsed-table collapsed_taxonomy_Seqs-all-2019_t10_silva_full_E-sampls

# Filter Cyanobacterial sequences for samples E
qiime feature-table filter-seqs
--i-data rep-seqs_Cyanobacteria_Seqs-all-2019_t10_silva_full_0.9.qza
--i-table table_Seqs-all-2019_t10_E.qza
--m-metadata-file metadata.tsv
--o-filtered-data filtered_E-sampls

# Filter of Cyanobacterial sequences
qiime feature-table filter-seqs
--i-data rep-seqs_Cyanobacteria_Seqs-all-2019_t10_silva_full_0.9.qza --i-table table_Seqs-all-2019_t10_E.qza
--m-metadata-file metadata.tsv
--o-filtered-data filtered_E-sampls
```

Filter samples
qiime feature-table filter-seqs
--i-data rep-seqs_Cyanobacteria_Seqs-all-2019_t10_silva_full_0.9.qza
--i-table table_Seqs-all-2019_t10_E.qza
--o-filtered-data filtered_E-sampls

Assign taxonomy using Silva 132 99% OTUs for samples E
qiime feature-classifier classify-sklearn
--i-classifier silva-132-99-nb-classifier.qza
--i-reads filtered_E-sampls.qza
--o-classification taxonomy_Seqs_t10_E-samples
